# Crystal structure of langbeinite-related Rb_0.743_K_0.845_Co_0.293_Ti_1.707_(PO_4_)_3_

**DOI:** 10.1107/S2056989015001826

**Published:** 2015-02-07

**Authors:** Nataliia Yu. Strutynska, Marina A. Bondarenko, Ivan V. Ogorodnyk, Vyacheslav N. Baumer, Nikolay S. Slobodyanik

**Affiliations:** a Department of Inorganic Chemistry, Taras Shevchenko National University of Kyiv, 64/13, Volodymyrska Str., 01601 Kyiv, Ukraine; b STC "Institute for Single Crystals", NAS of Ukraine, 60 Lenin Ave., 61001 Kharkiv, Ukraine; c V. N. Karazin National University, 4, Svobody Square, 61001 Kharkiv, Ukraine

**Keywords:** crystal structure, high-temperature crystallization, langbeinite-type structure, three-dimensional framework

## Abstract

Potassium rubidium cobalt(II)/titanium(IV) tris­(orthophosphate), Rb_0.743_K_0.845_Co_0.293_Ti_1.707_(PO_4_)_3_, has been obtained using a high-temperature crystallization method. The obtained compound has a langbeinite-type structure. The three-dimensional framework is built up from mixed-occupied (Co/Ti^IV^)O_6_ octa­hedra (point group symmetry .3.) and PO_4_ tetra­hedra. The K^+^ and Rb^+^ cations are statistically distributed over two distinct sites (both with site symmetry .3.) in the large cavities of the framework. They are surrounded by 12 O atoms.

## Abstract 2

Single crystals of the langbeinite-related phosphate Rb_0.743_K_0.845_Co_0.293_Ti_1.707_(PO_4_)_3_ have been prepared by crystallization of high-temperature self-flux K_2_O–Rb_2_O–P_2_O_5_–TiO_2_–CoO.

## Chemical context   

Nowadays, there are a number of reports on the synthesis and investigation of langbeinite-related complex phosphates, which exhibit inter­esting properties such as magnetic (Ogorodnyk *et al.*, 2006 ▸), luminescence (Zhang *et al.*, 2013 ▸; Chawla *et al.*, 2013 ▸) and phase transitions (Hikita *et al.*, 1977 ▸). It should be noted that compounds with a langbeinite-type structure are prospects for use as a matrix for the storage of nuclear waste (Orlova *et al.*, 2011 ▸). Zaripov *et al.* (2009 ▸) and Ogorodnyk *et al.* (2007*a* ▸) proved that caesium can be introduced into the cavity of a langbeinite framework that can be used for the immobil­ization of ^137^Cs in an inert matrix for safe disposal.

A large number of compounds with a langbeinite framework based on a variety of different valence elements are known. Three major types of substitutions of the elements are known as well as their combinations. They are: metal substitution in octa­hedra, element substitution in anion tetra­hedra, and substitution of ions in cavities. Among these compounds, potassium-containing langbeinites are the most studied (Ogorodnyk *et al.*, 2006 ▸, 2007*b* ▸,*c* ▸; Norberg, 2002 ▸; Orlova *et al.*, 2003 ▸). However, several reports concerning phosphate langbeinites with Rb^+^ in the cavities of the framework are known: Rb_2_FeZr(PO_4_)_3_ (Trubach *et al.*, 2004 ▸), Rb_2_YbTi(PO_4_)_3_ (Gustafsson *et al.*, 2005 ▸) and Rb_2_TiY(PO_4_)_3_ (Gustafsson *et al.*, 2006 ▸).

Herein, the structure of Rb_0.743_K_0.845_Co_0.293_Ti_1.707_(PO_4_)_3_, potassium rubidium cobalt(II)/titanium(IV) tris­(ortho­phos­phate) is reported.

## Structural commentary   

**Figure 1:**
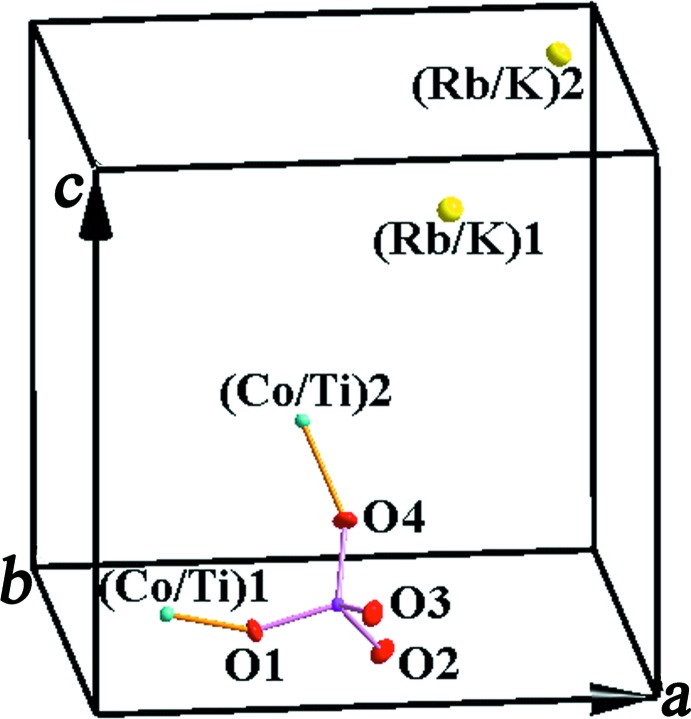
The asymmetric unit of Rb_0.743_K_0.845_Co_0.293_Ti_1.707_(PO_4_)_3_, showing displace­ment ellipsoids at the 50% probability level.

**Figure 2:**
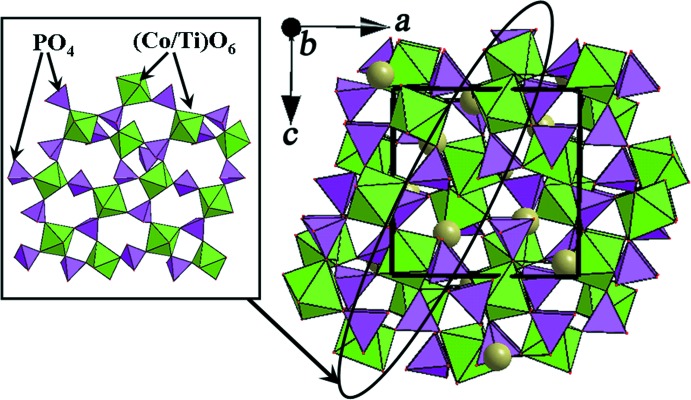
Two-dimensional net and three-dimensional framework for Rb_0.743_K_0.845_Co_0.293_Ti_1.707_(PO_4_)_3_.

The asymmetric unit of Rb_0.743_K_0.845_Co_0.293_Ti_1.707_(PO_4_)_3_ consists of two mixed-occupied (Co/Ti^IV^), two (Rb/K), one P and four oxygen positions (Fig. 1 ▸). The structure of the title compound is built up from mixed (Co/Ti^IV^)O_6_ octa­hedra and PO_4_ tetra­hedra, which are connected *via* common O-atom vertices. Each octa­hedron is linked to six adjacent tetra­hedra and reciprocally, each tetra­hedron is connected to four neighboring octa­hedra into a three-dimensional rigid framework (Fig. 2 ▸).

The oxygen environment of the metal atoms in the (Co/Ti^IV^)1O_6_ octa­hedra is slightly distorted, with *M*—O bonds of 1.940 (2) and 1.966 (2) Å. These distances are close to the corresponding bond lengths in K_2_Ti_2_(PO_4_)_3_ [*d*(Ti—O) = 1.877 (10)–1.965 (10) Å; Masse *et al.*, 1972 ▸], which could be explained by the small occupancy of cobalt in the mixed (Co/Ti^IV^)1 [occupancy = 0.1307 (9)] and (Co/Ti^IV^)2 [occupancy = 0.162 (3)] sites. It should be noted that (Co/Ti^IV^)2—O distances [1.949 (2) and 1.969 (2) Å] are slightly shorter than those in K_2_Co_0.5_Ti_1.5_(PO_4_)_3_ (Ogorodnyk *et al.*, 2006 ▸).

The orthophosphate tetra­hedra are also slightly distorted with P—O bond lengths ranging from 1.525 (2) to 1.531 (2) Å. These distances are almost identical to the corresponding ones in K_2_Co_0.5_Ti_1.5_(PO_4_)_3_ [*d*(P—O) =1.525 (2)–1.529 (9) Å; Ogorodnyk *et al.*, 2006 ▸). A comparison of the corresponding inter­atomic distances for the octa­hedra and tetra­hedra in Rb_0.743_K_0.845_Co_0.293_Ti_1.707_(PO_4_)_3_ and K_2_Co_0.5_Ti_1.5_(PO_4_)_3_ shows that partial substitution of K^+^ by Rb^+^ and decreasing the amount of cobalt slightly influences the distances in the polyhedra for Rb_0.743_K_0.845_Co_0.293_Ti_1.707_(PO_4_)_3_.

The K^+^ and Rb^+^ cations are located in large cavities of the three-dimensional framework in Rb_0.743_K_0.845_Co_0.293_Ti_1.707_(PO_4_)_3_. They are statistically distributed over two distinct sites in which they have partial occupancies of 0.540 (9) and 0.330 (18) for Rb1 and K1, respectively, and 0.203 (8) and 0.514 (17) for Rb2 and K2, respectively. For the determination of the (Rb/K)1 and (Rb/K)2 coordination numbers (CN), Voronoi–Dirichlet polyhedra (VDP) were built using the *DIRICHLET* program included in the *TOPOS* package (Blatov *et al.*, 1995 ▸). Analysis of the solid-angle (Ω) distribution revealed twelve (Rb/K)—O contacts for both the (Rb/K)1 and (Rb/K)2 sites (cut-off distance of 4.0 Å, neglecting those corresponding to Ω < 1.5%; Blatov *et al.*, 1998 ▸). The results of the construction of the Voronoi–Dirichlet polyhedra (Blatov *et al.*, 1995 ▸) indicated that the coordination scheme for (Rb/K)1 is described as [9 + 3] [nine meaning ‘ion–covalent’ bonds are in the range 2.896 (2)–3.095 (2) Å which have Ω > 5.0% and three (Rb/K)1—O distances equal to 3.438 (8) Å with Ω = 2.42%]. The (Rb/K)—O distances in the [(Rb/K)2O_12_]-polyhedra are in the range 2.870 (2)–3.219 (2) Å (4.91% < Ω < 9.5%).

The corresponding K1—O contacts in K_2_Co_0.5_Ti_1.5_(PO_4_)_3_ (Ogorodnyk *et al.*, 2006 ▸) are in the range 2.872 (2)–3.231 (3) Å while the K2—O distances in the K2O_12_ polyhedra are in the range 2.855 (2)–3.473 (3) Å, slightly longer than those in Rb_0.743_K_0.845_Co_0.293_Ti_1.707_(PO_4_)_3_. These results indicate that the substitution of K^+^ cations by Rb^+^ cations in Rb_0.743_K_0.845_Co_0.293_Ti_1.707_(PO_4_)_3_ caused a decrease of the (Rb/K)—O bond length. This fact confirms the rigidity of the framework and the suitability of the cavity dimensions to accommodate different sized ions whose size and nature insignificantly influence the framework.

## Synthesis and crystallization   

The title compound was prepared during crystallization of a self-flux in the Rb_2_O–K_2_O–P_2_O_5_–TiO_2_–CoO system. The starting components RbH_2_PO_4_ (4.0 g), KPO_3_ (2.4 g), TiO_2_ (0.532 g) and CoO (0.50 g) were ground in an agate mortar, placed in a platinum crucible and H_3_PO_4_ (85%, 0.42 g) was added. The mixture was heated up to 1273 K. The melt was kept at this temperature for one h. After that, the temperature was decreased to 873 K at a rate of 10 K h^−1^. The crystals of Rb_0.743_K_0.845_Co_0.293_Ti_1.707_(PO_4_)_3_ were separated from the rest flux by washing in hot water. The chemical composition of a single crystal was verified using EDX analysis. Analysis found: K 6.72, Rb 13.85, Co 3.74, Ti 16.86, P 19.96 and O 38.87 at%, while Rb_0.743_K_0.845_Co_0.293_Ti_1.707_(PO_4_)_3_ requires K 6.86, Rb 13.15, Co 3.60, Ti 17.06, P 19.36 and O 39.97 at%.

## Refinement   

Crystal data, data collection and structure refinement details are summarized in Table 1 ▸. The O-atom sites were determined from difference Fourier maps. It was assumed that both types of alkaline ions occupy cavity sites while the transition metals occupy framework sites. The occupancies were refined using linear combinations of free variables taking into account the total charge of the cell.

## Supplementary Material

Crystal structure: contains datablock(s) global, I. DOI: 10.1107/S2056989015001826/br2246sup1.cif

Structure factors: contains datablock(s) I. DOI: 10.1107/S2056989015001826/br2246Isup2.hkl

CCDC reference: 1045876

Additional supporting information:  crystallographic information; 3D view; checkCIF report

## Figures and Tables

**Table 1:** Experimental details

Crystal data	
Chemical formula	Rb_0.743_K_0.845_Co_0.293_Ti_1.707_(PO_4_)_3_
*M*_r_	480.40
Crystal system, space group	Cubic, *P*2_1_3
Temperature (K)	293
*a* ()	9.8527(1)
*V* (^3^)	956.46(2)
*Z*	4
Radiation type	Mo *K*
(mm^1^)	6.63
Crystal size (mm)	0.10 0.07 0.05

Data collection	
Diffractometer	Oxford Diffraction Xcalibur-3
Absorption correction	Multi-scan (Blessing, 1995 ▸)
*T*_min_, *T*_max_	0.559, 0.734
No. of measured, independent and observed [*I* > 2(*I*)] reflections	1414, 1414, 1312
*R*_int_	0.025
(sin /)_max_ (^1^)	0.804

Refinement	
*R*[*F* ^2^ > 2(*F* ^2^)], *wR*(*F* ^2^), *S*	0.026, 0.051, 1.05
No. of reflections	1414
No. of parameters	67
No. of restraints	3
_max_, _min_ (e ^3^)	0.37, 0.36
Absolute structure	Flack (1983 ▸), 612 Friedel pairs
Absolute structure parameter	0.024(10)

Computer programs: *CrysAlis CCD* and *CrysAlis RED* (Oxford Diffraction, 2006 ▸), *SHELXS97* and *SHELXL97* (Sheldrick, 2008 ▸), *DIAMOND* (Brandenburg, 1999 ▸), *WinGX* (Farrugia, 2012 ▸) and *enCIFer* (Allen *et al.*, 2004 ▸).

## supplementary crystallographic information

### Crystal data

**Table:**

Rb_0.743_K_0.845_Co_0.293_Ti_1.707_(PO_4_)_3_	*D*_x_ = 3.336 Mg m^−^^3^
*M**_r_* = 480.40	Mo *K*α radiation, λ = 0.71073 Å
Cubic, *P*2_1_3	Cell parameters from 1414 reflections
Hall symbol: P 2ac 2ab 3	θ = 2.9–34.9°
*a* = 9.8527 (1) Å	µ = 6.63 mm^−^^1^
*V* = 956.46 (2) Å^3^	*T* = 293 K
*Z* = 4	Tetrahedron, dark red
*F*(000) = 920	0.1 × 0.07 × 0.05 mm

### Data collection

**Table:**

Oxford Diffraction Xcalibur-3 diffractometer	1414 independent reflections
Radiation source: fine focus sealed tube	1312 reflections with *I* > 2σ(*I*)
Graphite monochromator	*R*_int_ = 0.025
φ and ω scans	θ_max_ = 34.9°, θ_min_ = 2.9°
Absorption correction: multi-scan (Blessing, 1995)	*h* = −10→10
*T*_min_ = 0.559, *T*_max_ = 0.734	*k* = 0→11
1414 measured reflections	*l* = 1→15

### Refinement

**Table:**

Refinement on *F*^2^	Secondary atom site location: difference Fourier map
Least-squares matrix: full	*w* = 1/[σ^2^(*F*_o_^2^) + (0.0183*P*)^2^] where *P* = (*F*_o_^2^ + 2*F*_c_^2^)/3
*R*[*F*^2^ > 2σ(*F*^2^)] = 0.026	(Δ/σ)_max_ = 0.051
*wR*(*F*^2^) = 0.051	Δρ_max_ = 0.37 e Å^−^^3^
*S* = 1.05	Δρ_min_ = −0.36 e Å^−^^3^
1414 reflections	Extinction correction: *SHELXL97* (Sheldrick, 2008)
67 parameters	Extinction coefficient: 0.0026 (6)
3 restraints	Absolute structure: Flack (1983), 612 Friedel pairs
Primary atom site location: structure-invariant direct methods	Absolute structure parameter: 0.024 (10)

### Special details

**Table:**

Geometry. All e.s.d.'s (except the e.s.d. in the dihedral angle between two l.s. planes) are estimated using the full covariance matrix. The cell e.s.d.'s are taken into account individually in the estimation of e.s.d.'s in distances, angles and torsion angles; correlations between e.s.d.'s in cell parameters are only used when they are defined by crystal symmetry. An approximate (isotropic) treatment of cell e.s.d.'s is used for estimating e.s.d.'s involving l.s. planes.
Refinement. Refinement of *F*^2^ against ALL reflections. The weighted *R*-factor *wR* and goodness of fit *S* are based on *F*^2^, conventional *R*-factors *R* are based on *F*, with *F* set to zero for negative *F*^2^. The threshold expression of *F*^2^ > σ(*F*^2^) is used only for calculating *R*-factors(gt) *etc*. and is not relevant to the choice of reflections for refinement. *R*-factors based on *F*^2^ are statistically about twice as large as those based on *F*, and *R*- factors based on ALL data will be even larger.

### Fractional atomic coordinates and isotropic or equivalent isotropic displacement parameters (Å^2^)

**Table:**

	*x*	*y*	*z*	*U*_iso_*/*U*_eq_	Occ. (<1)
Rb1	0.71155 (4)	0.71155 (4)	0.71155 (4)	0.02169 (18)	0.540 (9)
K1	0.71155 (4)	0.71155 (4)	0.71155 (4)	0.02169 (18)	0.330 (18)
Rb2	0.93045 (5)	0.93045 (5)	0.93045 (5)	0.0199 (3)	0.203 (8)
K2	0.93045 (5)	0.93045 (5)	0.93045 (5)	0.0199 (3)	0.514 (17)
Ti1	0.14135 (4)	0.14135 (4)	0.14135 (4)	0.00760 (12)	0.8693 (9)
Co1	0.14135 (4)	0.14135 (4)	0.14135 (4)	0.00760 (12)	0.1307 (9)
Ti2	0.41386 (3)	0.41386 (3)	0.41386 (3)	0.00709 (12)	0.838 (3)
Co2	0.41386 (3)	0.41386 (3)	0.41386 (3)	0.00709 (12)	0.162 (3)
P1	0.45604 (5)	0.22826 (5)	0.12582 (5)	0.00682 (10)	
O1	0.30739 (16)	0.23395 (16)	0.08086 (17)	0.0141 (3)	
O2	0.54329 (18)	0.29756 (17)	0.01814 (17)	0.0179 (3)	
O3	0.50157 (16)	0.08190 (16)	0.14744 (18)	0.0168 (3)	
O4	0.47835 (17)	0.30686 (19)	0.25786 (18)	0.0190 (4)	

### Atomic displacement parameters (Å^2^)

**Table:**

	*U*^11^	*U*^22^	*U*^33^	*U*^12^	*U*^13^	*U*^23^
Rb1	0.02169 (18)	0.02169 (18)	0.02169 (18)	0.00120 (13)	0.00120 (13)	0.00120 (13)
K1	0.02169 (18)	0.02169 (18)	0.02169 (18)	0.00120 (13)	0.00120 (13)	0.00120 (13)
Rb2	0.0199 (3)	0.0199 (3)	0.0199 (3)	−0.00172 (19)	−0.00172 (19)	−0.00172 (19)
K2	0.0199 (3)	0.0199 (3)	0.0199 (3)	−0.00172 (19)	−0.00172 (19)	−0.00172 (19)
Ti1	0.00760 (12)	0.00760 (12)	0.00760 (12)	0.00051 (11)	0.00051 (11)	0.00051 (11)
Co1	0.00760 (12)	0.00760 (12)	0.00760 (12)	0.00051 (11)	0.00051 (11)	0.00051 (11)
Ti2	0.00709 (12)	0.00709 (12)	0.00709 (12)	−0.00033 (11)	−0.00033 (11)	−0.00033 (11)
Co2	0.00709 (12)	0.00709 (12)	0.00709 (12)	−0.00033 (11)	−0.00033 (11)	−0.00033 (11)
P1	0.0059 (2)	0.0076 (2)	0.0070 (2)	−0.00019 (16)	0.00100 (16)	−0.00054 (17)
O1	0.0088 (7)	0.0167 (8)	0.0169 (8)	0.0001 (5)	−0.0032 (6)	0.0021 (6)
O2	0.0183 (9)	0.0185 (8)	0.0170 (8)	0.0001 (7)	0.0075 (6)	0.0049 (6)
O3	0.0160 (8)	0.0130 (8)	0.0214 (8)	0.0064 (6)	0.0022 (6)	0.0034 (6)
O4	0.0196 (9)	0.0229 (10)	0.0146 (8)	−0.0027 (7)	0.0001 (7)	−0.0099 (6)

### Geometric parameters (Å, º)

**Table:**

Rb1—O1^i^	2.8956 (17)	Rb2—O4^ix^	3.219 (2)
Rb1—O1^ii^	2.8956 (17)	Rb2—O4^viii^	3.219 (2)
Rb1—O1^iii^	2.8956 (17)	Ti1—O2^x^	1.9404 (17)
Rb1—O2^iv^	3.0780 (19)	Ti1—O2^xi^	1.9404 (17)
Rb1—O2^v^	3.0780 (19)	Ti1—O2^xii^	1.9404 (17)
Rb1—O2^vi^	3.0780 (19)	Ti1—O1^xiii^	1.9657 (16)
Rb1—O4^iv^	3.0945 (18)	Ti1—O1	1.9657 (16)
Rb1—O4^vi^	3.0945 (18)	Ti1—O1^xiv^	1.9657 (16)
Rb1—O4^v^	3.0945 (18)	Ti2—O3^ii^	1.9494 (16)
Rb2—O3^iv^	2.8703 (18)	Ti2—O3^iii^	1.9494 (16)
Rb2—O3^vi^	2.8703 (18)	Ti2—O3^i^	1.9494 (16)
Rb2—O3^v^	2.8703 (18)	Ti2—O4^xiii^	1.9691 (17)
Rb2—O2^vii^	2.9452 (19)	Ti2—O4	1.9691 (17)
Rb2—O2^viii^	2.9452 (19)	Ti2—O4^xiv^	1.9691 (17)
Rb2—O2^ix^	2.9452 (19)	P1—O3	1.5252 (17)
Rb2—O4^iv^	3.028 (2)	P1—O2	1.5266 (17)
Rb2—O4^vi^	3.028 (2)	P1—O4	1.5299 (17)
Rb2—O4^v^	3.028 (2)	P1—O1	1.5312 (16)
Rb2—O4^vii^	3.219 (2)		
			
O1^i^—Rb1—O1^ii^	90.95 (5)	O2^ix^—Rb2—O4^v^	94.29 (5)
O1^i^—Rb1—O1^iii^	90.95 (5)	O4^iv^—Rb2—O4^v^	87.83 (5)
O1^ii^—Rb1—O1^iii^	90.95 (5)	O4^vi^—Rb2—O4^v^	87.83 (5)
O1^i^—Rb1—O2^iv^	145.70 (5)	O3^iv^—Rb2—O4^vii^	86.01 (4)
O1^ii^—Rb1—O2^iv^	82.60 (4)	O3^vi^—Rb2—O4^vii^	55.94 (4)
O1^iii^—Rb1—O2^iv^	55.72 (4)	O3^v^—Rb2—O4^vii^	157.18 (5)
O1^i^—Rb1—O2^v^	55.72 (4)	O2^vii^—Rb2—O4^vii^	46.55 (5)
O1^ii^—Rb1—O2^v^	145.70 (5)	O2^viii^—Rb2—O4^vii^	86.90 (5)
O1^iii^—Rb1—O2^v^	82.60 (4)	O2^ix^—Rb2—O4^vii^	101.29 (5)
O2^iv^—Rb1—O2^v^	119.386 (9)	O4^iv^—Rb2—O4^vii^	53.03 (6)
O1^i^—Rb1—O2^vi^	82.60 (4)	O4^vi^—Rb2—O4^vii^	104.695 (9)
O1^ii^—Rb1—O2^vi^	55.72 (4)	O4^v^—Rb2—O4^vii^	137.38 (4)
O1^iii^—Rb1—O2^vi^	145.70 (5)	O3^iv^—Rb2—O4^ix^	55.94 (4)
O2^iv^—Rb1—O2^vi^	119.386 (9)	O3^vi^—Rb2—O4^ix^	157.18 (5)
O2^v^—Rb1—O2^vi^	119.386 (9)	O3^v^—Rb2—O4^ix^	86.01 (4)
O1^i^—Rb1—O4^iv^	165.33 (5)	O2^vii^—Rb2—O4^ix^	86.90 (5)
O1^ii^—Rb1—O4^iv^	82.87 (5)	O2^viii^—Rb2—O4^ix^	101.29 (5)
O1^iii^—Rb1—O4^iv^	102.41 (5)	O2^ix^—Rb2—O4^ix^	46.55 (5)
O2^iv^—Rb1—O4^iv^	46.75 (5)	O4^iv^—Rb2—O4^ix^	104.695 (9)
O2^v^—Rb1—O4^iv^	131.42 (5)	O4^vi^—Rb2—O4^ix^	137.38 (4)
O2^vi^—Rb1—O4^iv^	82.92 (5)	O4^v^—Rb2—O4^ix^	53.03 (6)
O1^i^—Rb1—O4^vi^	82.87 (5)	O4^vii^—Rb2—O4^ix^	115.47 (2)
O1^ii^—Rb1—O4^vi^	102.41 (5)	O3^iv^—Rb2—O4^viii^	157.18 (5)
O1^iii^—Rb1—O4^vi^	165.33 (5)	O3^vi^—Rb2—O4^viii^	86.01 (4)
O2^iv^—Rb1—O4^vi^	131.42 (5)	O3^v^—Rb2—O4^viii^	55.94 (4)
O2^v^—Rb1—O4^vi^	82.92 (5)	O2^vii^—Rb2—O4^viii^	101.29 (5)
O2^vi^—Rb1—O4^vi^	46.75 (5)	O2^viii^—Rb2—O4^viii^	46.55 (5)
O4^iv^—Rb1—O4^vi^	85.47 (6)	O2^ix^—Rb2—O4^viii^	86.90 (5)
O1^i^—Rb1—O4^v^	102.41 (5)	O4^iv^—Rb2—O4^viii^	137.38 (4)
O1^ii^—Rb1—O4^v^	165.33 (5)	O4^vi^—Rb2—O4^viii^	53.03 (6)
O1^iii^—Rb1—O4^v^	82.87 (5)	O4^v^—Rb2—O4^viii^	104.695 (9)
O2^iv^—Rb1—O4^v^	82.92 (5)	O4^vii^—Rb2—O4^viii^	115.47 (2)
O2^v^—Rb1—O4^v^	46.75 (5)	O4^ix^—Rb2—O4^viii^	115.47 (2)
O2^vi^—Rb1—O4^v^	131.42 (5)	O2^x^—Ti1—O2^xi^	90.14 (7)
O4^iv^—Rb1—O4^v^	85.47 (6)	O2^x^—Ti1—O2^xii^	90.14 (7)
O4^vi^—Rb1—O4^v^	85.47 (6)	O2^xi^—Ti1—O2^xii^	90.14 (7)
O3^iv^—Rb2—O3^vi^	101.27 (5)	O2^x^—Ti1—O1^xiii^	91.43 (7)
O3^iv^—Rb2—O3^v^	101.27 (5)	O2^xi^—Ti1—O1^xiii^	88.09 (7)
O3^vi^—Rb2—O3^v^	101.27 (5)	O2^xii^—Ti1—O1^xiii^	177.64 (7)
O3^iv^—Rb2—O2^vii^	99.30 (5)	O2^x^—Ti1—O1	88.09 (7)
O3^vi^—Rb2—O2^vii^	96.75 (5)	O2^xi^—Ti1—O1	177.64 (7)
O3^v^—Rb2—O2^vii^	149.30 (5)	O2^xii^—Ti1—O1	91.43 (7)
O3^iv^—Rb2—O2^viii^	149.30 (5)	O1^xiii^—Ti1—O1	90.39 (7)
O3^vi^—Rb2—O2^viii^	99.30 (5)	O2^x^—Ti1—O1^xiv^	177.64 (7)
O3^v^—Rb2—O2^viii^	96.75 (5)	O2^xi^—Ti1—O1^xiv^	91.43 (7)
O2^vii^—Rb2—O2^viii^	55.61 (6)	O2^xii^—Ti1—O1^xiv^	88.09 (7)
O3^iv^—Rb2—O2^ix^	96.75 (5)	O1^xiii^—Ti1—O1^xiv^	90.39 (7)
O3^vi^—Rb2—O2^ix^	149.30 (5)	O1—Ti1—O1^xiv^	90.39 (7)
O3^v^—Rb2—O2^ix^	99.30 (5)	O3^ii^—Ti2—O3^iii^	91.87 (7)
O2^vii^—Rb2—O2^ix^	55.61 (6)	O3^ii^—Ti2—O3^i^	91.87 (7)
O2^viii^—Rb2—O2^ix^	55.61 (6)	O3^iii^—Ti2—O3^i^	91.87 (7)
O3^iv^—Rb2—O4^iv^	49.64 (5)	O3^ii^—Ti2—O4^xiii^	94.30 (7)
O3^vi^—Rb2—O4^iv^	52.65 (5)	O3^iii^—Ti2—O4^xiii^	172.63 (8)
O3^v^—Rb2—O4^iv^	116.41 (6)	O3^i^—Ti2—O4^xiii^	83.90 (7)
O2^vii^—Rb2—O4^iv^	94.29 (5)	O3^ii^—Ti2—O4	83.90 (7)
O2^viii^—Rb2—O4^iv^	138.73 (5)	O3^iii^—Ti2—O4	94.30 (7)
O2^ix^—Rb2—O4^iv^	133.42 (5)	O3^i^—Ti2—O4	172.63 (8)
O3^iv^—Rb2—O4^vi^	116.41 (6)	O4^xiii^—Ti2—O4	90.39 (7)
O3^vi^—Rb2—O4^vi^	49.64 (5)	O3^ii^—Ti2—O4^xiv^	172.63 (8)
O3^v^—Rb2—O4^vi^	52.65 (5)	O3^iii^—Ti2—O4^xiv^	83.90 (7)
O2^vii^—Rb2—O4^vi^	133.42 (5)	O3^i^—Ti2—O4^xiv^	94.30 (7)
O2^viii^—Rb2—O4^vi^	94.29 (5)	O4^xiii^—Ti2—O4^xiv^	90.39 (7)
O2^ix^—Rb2—O4^vi^	138.73 (5)	O4—Ti2—O4^xiv^	90.39 (7)
O4^iv^—Rb2—O4^vi^	87.83 (5)	O3—P1—O2	110.75 (9)
O3^iv^—Rb2—O4^v^	52.65 (5)	O3—P1—O4	108.52 (11)
O3^vi^—Rb2—O4^v^	116.41 (6)	O2—P1—O4	106.48 (10)
O3^v^—Rb2—O4^v^	49.64 (5)	O3—P1—O1	110.87 (9)
O2^vii^—Rb2—O4^v^	138.73 (5)	O2—P1—O1	108.74 (10)
O2^viii^—Rb2—O4^v^	133.42 (5)	O4—P1—O1	111.40 (10)

Symmetry codes: (i) −*z*+1/2, −*x*+1, *y*+1/2; (ii) *y*+1/2, −*z*+1/2, −*x*+1; (iii) −*x*+1, *y*+1/2, −*z*+1/2; (iv) −*x*+3/2, −*y*+1, *z*+1/2; (v) *z*+1/2, −*x*+3/2, −*y*+1; (vi) −*y*+1, *z*+1/2, −*x*+3/2; (vii) −*y*+3/2, −*z*+1, *x*+1/2; (viii) −*z*+1, *x*+1/2, −*y*+3/2; (ix) *x*+1/2, −*y*+3/2, −*z*+1; (x) −*y*+1/2, −*z*, *x*−1/2; (xi) −*z*, *x*−1/2, −*y*+1/2; (xii) *x*−1/2, −*y*+1/2, −*z*; (xiii) *y*, *z*, *x*; (xiv) *z*, *x*, *y*.
